# Dipole instability after an ultrashort XUV pulse in N_2_: population inversion and timescales

**DOI:** 10.1038/s41598-025-27507-7

**Published:** 2025-12-12

**Authors:** Dale Hughes, Daniel Dundas, Phuong Mai Dinh, Paul-Gerhard Reinhard, Eric Suraud

**Affiliations:** 1 School of Mathematics and Physics, Queen’s University Belfast, University Road, BT7 1NN Belfast, UK; 2 Laboratoire de Physique Théorique, University of Toulouse and CNRS, 31062 Toulouse Cedex, France; 3 Friedrich-Alexander Universität, 91054 Erlangen, Germany

**Keywords:** Atomic and molecular interactions with photons, Attosecond science

## Abstract

We study the response of N_2_ to a range of XUV laser pulses using real-time time-dependent density functional theory on real-space grids in order to better understand and quantify the internal processes that lead to dipole instabilities. These instabilities, documented in a series of recent papers, develop following the generation of a population inversion in the molecule, in this case induced by the laser pulse. In the current paper, a series of laser pulses having durations of one femtosecond are considered and we explore how the growth rate, total ionization and amount of population inversion changes. This reveals a new aspect to the instability in which the growth rate starts decreasing with increasing pulse intensity. In addition, by using a range of different pseudopotential descriptions of the electron-ion interactions, we find that the observed behaviour is qualitatively independent of the pseudopotential used.

## Introduction

Ultrashort laser pulses^1,2^, now exploring the XUV to X-ray frequency regime, are increasingly becoming fashionable, which triggers an impressive development of more and more detailed investigations of ultrafast processes in molecules and clusters^3,4^. Accessing very short pulse durations in the attosecond regime, together with the great flexibility in pulse shaping, should allow access to fully time-resolved measurements down to the electronic timescale^5^. High photon energies furthermore allow one to explore the impact of direct electronic excitations in deeply lying states. This triggers various relaxation processes such as, for example, Auger decay ^6^, shake-up ^7^ and shake-off ^6^ processes or charge migration in covalent molecules ^8^. One can also mention here interatomic (or intermolecular) Coulomb decay ^9^, or giant autoionization resonances in high harmonic generation ^10^. Clearly the field opens up promising research directions to understand a multitude of dynamical scenarios from atoms to clusters and molecules^11^.

From the theoretical side, the highly dynamical, potentially far off equilibrium, nature of ultrafast scenarios make fully detailed treatments of excitation and relaxation extremely demanding^12,13^. Modelling can be simplified by assuming an instantaneous removal of a, possibly even deeply, bound electron by the ultrafast pulse^14^. This major simplification overlooks details of the excitation process, but allows an arbitrary choice of the target hole. Such an approach has, for example, been widely used to explore charge migration and transfer in molecular systems^15–19^.

In a recent series of papers aiming at investigating in some detail the excitation processes induced by a short XUV pulse, we identified an unexpected scenario characterized by the development of large amplitude dipole oscillations^20–22^. Once irradiation by a far-off resonance laser pulse is over, the dipole signal normally shrinks down to zero. Total ionization rapidly levels off and stays constant. For a certain range of laser frequencies we observe that after some time the dipole increases again to reach sizable values, thus generating extra ionization. The effect can be measured on time-resolved Photo-Electron Spectra (PES) with the delayed appearance of an extra low energy peak, which could be used for experimental identification of the process. As well as observing these dipole oscillations in cases where we remove an electron from a deeply lying valence state ^20^, we have also see similar oscillations for single particle excitations, for example, when moving an electron in the highest-occupied molecular orbital (HOMO) to the lowest-unoccupied molecular orbital (LUMO). We presently call this phenomenon a dipole instability as it cannot be mapped to well known deexcitation mechanisms such as Auger decay or shake-up/shake-off processes. Additionally, it has been shown by comparing various numerical approaches that this instability is not a numerical problem.

However, it is not yet excluded that it is a defect of the level of theory used to describe electrons. Briefly, we describe electronic structure using Time-Dependent Density Functional Theory (TDDFT) ^23^. Exchange-correlation is described at the Local Density Approximation (LDA) level incorporating the Perdew-Wang parameterization of the correlation functional ^24^. The primary actual level of description is thus Time Dependent Local Density Approximation, TDLDA. But it is well-known that functionals such as LDA contain self-interaction errors which means that the long range behaviour of the exchange-correlation potential is exponential instead of Coulombic. This means that electrons are too loosely bound and excited states are not accurately described ^25^. Therefore, we supplement the LDA functional with the Average Density Self-Interaction Correction (ADSIC) ^26–29^, which reinstates the correct long-range Coulombic behaviour in an orbital-independent fashion. We can also go beyond standard TDLDA+ADSIC to include incoherent dynamical correlations from electron-electron collisions using a Relaxation Time Ansatz (RTA) ^30^ and find that including such correlations does not suppress the instability ^22^.

In the original studies of these instabilities, several excitation mechanisms were considered, ranging from instantaneous hole creation to the interaction of the molecules with short duration laser pulses. A key parameter which triggers the instability is the laser frequency. Low-frequency pulses skim electrons from the Fermi surface, while deeper lying electron states come naturally and increasingly into play with increasing laser frequency^31,32^. The result is an equi-distribution of depletion among the valence states, as has been seen in Na clusters ^31,33^ or C$_{60}~$^32^.

For properly tuned XUV pulses, with durations around 1 fs, we can deliver an ionization mechanism very close to an instantaneous deep hole creation^21^ (indeed it somewhat justifies the simplified excitation model of an instantaneous hole creation^20^). The creation of this deep hole leads to a population inversion: energy can be released by filling the deep lying hole with a much less bound electron. This population inversion looks like the source of the delayed reappearance of the dipole signal well after the laser pulse and the original dipole are over. We note that while such short-duration laser pulses are currently difficult to obtain experimentally, we have also observed instabilities using pulses with durations and intensities that are more easily attainable ^22^. In that case, the dynamical response becomes more complicated as the ionic dynamics become important.

The aim of this paper is to understand and quantify the onset of these dipole instabilities in more detail. Using an exemplar system, namely N_2_ irradiated by ultrafast (1 fs), linearly polarized laser pulses, we will study how key quantities (such as the rate of growth of the dipole and the amount population inversion produced) change as we systematically vary the laser intensity, wavelength and the orientation of the laser polarization direction with the molecular axis. We will also consider how different descriptions of the electron-ion interactions impact the instabilities by using different pseudopotentials to describe the electron-ion coupling ^34,35^. In order to simplify the description, we shall restrict the theory at the level of standard TDLDA (with ADSIC but without RTA) and keep ions frozen.

## Computational details

### The dynamical description through the Kohn–Sham equations

The calculations presented in this paper have been carried out using two separate computer packages, QDD (Quantum Dissipative Dynamics) ^36^ and EDAMAME (Ehrenfest DynAMics on Adaptive MEshes) ^37^, both of which solve the time-dependent Kohn-Sham (TDKS) equations of TDDFT, but using different numerical approaches. Both packages use pseudopotentials (described later) to replace the effect of the core electrons meaning that for N$_2$ we consider $N_e=10$ electrons. In that case we write the time-dependent electron density in terms of $N_e$ TDKS orbitals, $\psi _{j} (\textbf{r},t)$, as

1 $$\begin{aligned} \rho (\textbf{r},t) = \sum _{j=1}^{N_e} \left| \psi _{j } (\textbf{r},t) \right| ^2, \end{aligned}$$

where we neglect for simplicity the spin degree-of-freedom in this notation. In this case orbitals 1–5 denote our spin-up electrons while orbitals 6–10 denote the spin-down electrons. For the simulations we will consider that the response of both spin components will be identical. The orbitals are propagated by the TDKS equations (in atomic units):

2 $$\begin{aligned} i\frac{\partial }{\partial t}\psi _{j} (\textbf{r},t) = \Biggr [&-\frac{1}{2} \nabla ^2 + V_{\text{H}}(\textbf{r}, t) + V_{\text{ext}}(\textbf{r}, \textbf{R}_1,\textbf{R}_2,t) + V_{\text{xc}}(\textbf{r}, t) \Biggr ] \psi _{j} (\textbf{r},t) \quad\quad\quad\quad j = 1,\dots , N_e \end{aligned}$$

where $\textbf{r}$ denotes the electronic position vector while $\textbf{R}_1$ and $\textbf{R}_2$ denote the positions of the two ions in N$_2$, an ion being a nucleus plus the core electrons not described by TDDFT. In all the calculations except those presented in Fig. 8, the ions are considered frozen and the molecule is aligned along the *z*-axis. In Eq. (2), $V_{\text{H}}(\textbf{r}, t)$ is the Hartree potential, $V_{\text{ext}}(\textbf{r}, \textbf{R}_1, \textbf{R}_2, t)$ is the external potential, and $V_{\text{xc}}(\textbf{r}, t)$ is the exchange-correlation potential which is approximated using the LDA incorporating the Perdew-Wang parameterization of the correlation functional ^24^. This functional is supplemented with the average density self-interaction correction (ADSIC) ^26–29^ which reinstates the correct long-range behaviour in an orbital-independent fashion to give a realistic description of single particle energies.

The external potential accounts for both electron-ion interactions and the interaction of the laser field with the electrons and can be written as

3 $$\begin{aligned} V_{\text{ext}}(\textbf{r}, \textbf{R}_1, \textbf{R}_2, t) = V_{\text{ions}}(\textbf{r}, \textbf{R}_1, \textbf{R}_2, t) + V_{\text{pulse}}(\textbf{r}, t). \end{aligned}$$

Working within the dipole approximation and considering a linearly-polarised laser pulse aligned along the *z*-axis, the interaction of the laser field with the electrons is written as

4a $$\begin{aligned} V_\textrm{pulse}(\textbf{r}, t) = e{E}_0z\,f(t) \cos \left[ \omega _\textrm{pulse}\left( t-\frac{T_\textrm{pulse}}{2}\right) \right] \end{aligned}$$

where

4b $$\begin{aligned} f(t) = \left\{ \begin{array}{ll} \sin ^2{\left( \displaystyle \frac{\pi t}{T_\textrm{pulse}}\right) } & t\in \{0,\, T_\textrm{pulse}\} \\ 0 & \text{ otherwise } \end{array}\right. . \end{aligned}$$

The pulse parameters are: frequency $\omega _\textrm{pulse}$, duration $T_\textrm{pulse}$, and field strength ${E}_0$ (related to the pulse intensity as $I_\textrm{pulse} \propto {E}_0^2$).

### Pseudopotential treatment of electron-ion interactions.

The electron-ion interactions, $V_{\text{ions}}(\textbf{r}, \textbf{R}_1, \textbf{R}_2, t)$, are described using pseudopotentials. QDD uses Goedecker-type pseudopotentials^34^ while EDAMAME uses Troullier-Martins (TM) pseudopotentials ^35^ in the Kleinman-Bylander form ^38^. The TM pseudopotentials were generated using the atomic pseudopotential engine ^39^ with the cut-off radii for 2s and 2p electrons set to 1.4 a$_0$. For the Goedecker pseudopotentials, we use two versions. The first version (denoted as ‘G’) uses standard Goedecker parameters: core radii 0.289 a$_0$ for the local part and 0.257 a$_0$ for the non-local part  ^34^. Both radii are somewhat smaller than 0.3 a$_0$ and so this pseudopotential is rather hard. For a more friendly numerical representation on a coordinate-space grid, our second version (denoted as ‘sG’) retunes the parameters (0.5 a$_0$ for both the local and non-local terms) to produce a slightly softer pseudopotential.

### EDAMAME calculation details

EDAMAME solves the TDKS equations using adaptive real-space grid techniques in 3D ^37^. In the calculations presented in this paper, a global adaption is used where the position vector on the grid is scaled to give a regular grid spacing that doubles when going from the inner to the outer region ^22^. The transition point is set to 10 $a_0$ for all coordinates with the inner region grid spacings set to 0.2 a$_0$ (i.e. increasing to 0.4 a$_0$ in the outer region). Along each coordinate we use 275 grid points which gives a grid extent of $\pm 44.8$ a$_0$. All differential operators in the TDKS equations are approximated using 5-point central differences. Stationary electronic states are computed using a Chebyshev Filtered Subspace Iteration method ^40^. The TDKS equations are then propagated using an 18th-order Arnoldi propagation scheme with a time step of 0.00121 fs  ^37^. This scheme is supplemented by the predictor-corrector scheme to improve energy conservation ^36^. Electron emission is described via absorbing boundary conditions using a mask function ^37^.

### QDD calculation details

Quantum Dissipative Dynamics (QDD) ^36^ also solves the TDKS equations on a real-space grid using the real-time approach. Here we adopt a Fourier-Transform based method for evaluation of kinetic energy terms during time propagation, where the TV-splitting technique is employed ^41^. The employed numerical parameters are converged separately for each Goedecker-type pseudopotential. Calculations with the sG form use a uniform grid spacing of 0.588705 a$_0$, with 128 grid points, giving a box size of $\pm 37.7$ a$_0$ in all dimensions. We then use a time step of 0.000605 fs for time propagation. When using the *G* pseudopotential, we must use a grid spacing of 0.3 a$_0$, and increase to 180 grid points to get a box size of $\pm 27.0$ a$_0$ in all dimensions. Due to the resultant higher Fourier components present on the finer grid, we must use a time step of 0.000242 fs.

### Comparisons of ground-state properties

Using the parameters detailed above, geometry relaxation is carried out to obtain the equilibrium bond length. For EDAMAME, we obtain a bond length of 1.99 a$_0$ while both Goedecker pseudopotentials (G and sG) produce the same bond length of 2.02 a$_0$. These compare well with the experimental bond length of 2.0749 a$_0$ ^42^. Using these bond lengths, single-particle energies of the Kohn-Sham orbitals are presented in Table 1.

**Table 1:** Single-particle energies of the 10 valence electrons of N$_2$ computed in QDD with the G and sG pseudopotentials, and in EDAMAME with the TM pseudopotential (see text for details). Orbitals 1–5 denote spin-up electrons while orbitals 6–10 denote spin-down electrons.

Orbital		Orbital energy (eV)		
index		G	sG	TM
1,	6	–34.802	–33.028	–34.901
2,	7	–18.404	–18.587	–18.194
3,	8	–17.773	–17.984	–18.097
4,	9	–17.773	–17.984	–18.097
5,	10	–15.638	–15.206	–15.583

**Fig. 1:**
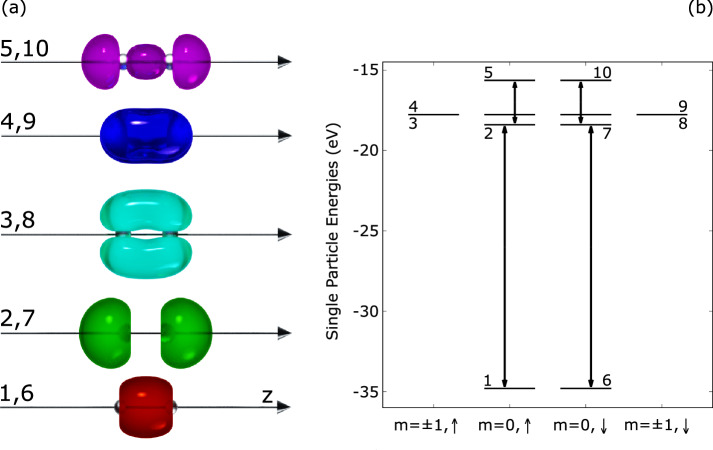
Left, (**a**): isosurface renderings of the charge density of the 10 ground state Kohn-Sham orbitals of N$_2$. Orbitals 1–5 denote the spin-up orbitals while orbitals 6–10 denote the spin-down orbitals. The Kohn-Sham orbitals are calculated using TM pseudopotentials in EDAMAME with the molecular axis aligned along the *z*-axis. Right, (**b**) ground state single-particle spectrum of N$_2$ calculated in EDAMAME. For better visibility, the orbitals are separated according to azimuthal angular momentum *m* and spin. The orbital labels are the same as in the left panel. The vertical arrows indicate the non-vanishing transition dipole moments in the *z* direction.

Figure 1 depicts the charge density of each occupied orbital and emphasizes the non-vanishing transition dipole moments along the molecular axis, that is, the coupling between orbitals 2 and 5 (and similarly between orbitals 7 and 10) on the one hand, and between orbitals 1 and 2 (and similarly between orbitals 6 and 7) on the other hand.

### Observables

The population of the Kohn-Sham orbital $\psi _j(\textbf{r},t)$ is defined by

5 $$\begin{aligned} P_j(t)=\langle \psi _{j}(\textbf{r},t) | \psi _{j}(\textbf{r},t) \rangle \end{aligned}$$

and its complement, the depletion by

6 $$\begin{aligned} \overline{P}_j(t)=1-P_j(t). \end{aligned}$$

Initially, an occupied orbital is completely filled, i.e. $P_j(0)=1$. Electron loss through the absorbing boundary conditions leads to a steady decrease of population and a corresponding increase of depletion. The total depletion, summed over all occupied orbitals, gives the total ionization

7 $$\begin{aligned} I(t)=\sum _{j=1}^{N_e}\overline{P}_j(t) \;. \end{aligned}$$

To quantify the content of the initially occupied Kohn-Sham orbital $\psi _n(\textbf{r},0)$ in the system at later times we calculate the initial orbital overlaps

8 $$\begin{aligned} S_n(t) = \sum _{j=1}^{N_e} \langle \psi _n(\textbf{r},0) | \psi _j(\textbf{r},t) \rangle \langle \psi _j(\textbf{r},t) | \psi _n(\textbf{r},0) \rangle \hspace{2cm}n = 1, \dots , N_e. \end{aligned}$$

Of course, $S_n(0)=1$ for all initial orbitals and

9 $$\begin{aligned} \displaystyle \sum _{n=1}^{N_e} S_n(0)=N_e=10. \end{aligned}$$

Later on, as the TDKS orbitals depart from the initial orbitals, one expects

10 $$\begin{aligned} \sum _{n=1}^{N_e} S_n(t)<N_e. \end{aligned}$$

Naturally, these orbital overlaps will not necessarily capture the full time-evolving character of the response. An alternative approach would be to project the time-evolving Kohn-Sham orbitals onto the instantaneous eigenstates of the Kohn-Sham Hamiltonian. However, such an approach is computationally very expensive and so we have tested it in our 2D model ^21^ and shown that both approaches provide similar results for the laser parameters considered in this paper.

Given that the presence of deeply lying hole is a prerequisite to the emergence of the dipole instability, we further define a population inversion observable

11 $$\begin{aligned} \Delta _{12}(t) = 2 \Bigr (P_2(t) - P_1(t) \Bigr ) \end{aligned}$$

in terms of the populations of orbitals 1 and 2, including the spin factor of 2. We specifically consider those two orbitals as the transition dipole moment in the *z* direction between them is non-vanishing, as shown in Fig. 1. In addition, orbital 1 (equivalently orbital 6) interacts most strongly with the laser pulse in the regime of interest. These factors combine to create a strong coupling between these two orbitals.

We will finally consider the electronic dipole moment. In atomic units, it reads

12 $$\begin{aligned} \textbf{d}(t) = \sum _{j=1}^{N_e} \int d^3 \textbf{r}\ \psi _j^*(\textbf{r},t) \ \textbf{r}\ \psi _j(\textbf{r},t) = d_x(t)\textbf{i} + d_y(t)\textbf{j} +d_z(t)\textbf{k}. \end{aligned}$$

While we will consider specific components of the dipole moment later, we mainly consider the magnitude of the dipole moment

13 $$\begin{aligned} \left| \textbf{d}(t)\right| =\sqrt{d_x^2(t) + d_y^2(t) + d_z^2(t)}. \end{aligned}$$

The latter has the advantage that we spot any dipole signal independent of the spatial direction. However, for all the calculations considered here in which the laser polarization direction is aligned along the molecular axis, we find that only $d_z(t)$ shows a significant response.

## Results

### Illustration and quantification of the dipole instability

**Fig. 2:**
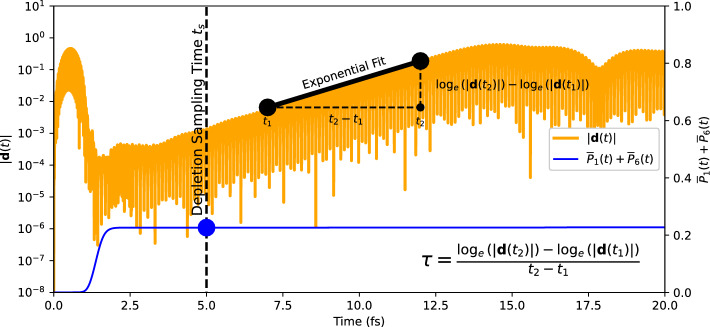
Evolution of the dipole signal (orange line) after excitation of N$_2$ by an XUV pulse with frequency $\omega _\textrm{pulse}=59.04$ eV, intensity $I\textrm{pulse}=$ $1\times 10^{15} \text{ W/cm}^{2}$ and pulse length $T_\textrm{pulse}=1$ fs shown on a logarithmic scale, computed with TM pseudopotentials in EDAMAME. Both the laser polarization direction and the molecular axis are aligned along the *z*-axis. The blue line shows the depletion, $\overline{P}_1(t)+\overline{P}_6(t)$, of the two lowest orbitals 1 and 6, see Eq. (6). The vertical dashes indicates the time $t_s$ at which the “final” depletion of orbitals 1 and 6 is read off (see *y*-scale to the right), to exclude further possible further ionization due to the instability itself. The heavy black line complemented by black dots and dashed lines illustrates how we calculate the growth time, $\tau$, and the growth rate, $1/\tau$, of the instability (see text for details).

Figure 2 illustrates the typical time evolution of a dipole instability after a short XUV pulse. A logarithmic scale on the *y*-axis clearly illustrates the exponential growth of the dipole (linear in that log scale). The initial peak in the dipole moment around 0.5 fs reflects the immediate response to the external electromagnetic field. The laser frequency is far enough away from any resonances and so the dipole signal drops to near zero immediately after the pulse. But subsequently, the dipole oscillations return, growing exponentially until the signal saturates.

As discussed in previous publications^20,21^, the dipole instability is driven by a population inversion which in the present case is generated by a properly chosen XUV pulse. The blue line in Fig. 2 shows the total depletion of the two lowest orbitals 1 and 6. The much higher-lying orbitals 2 and 7 remain significantly less depleted, and they are connected to orbitals 1 and 6 respectively by strong dipole transitions. In the ideal case of an instantaneous depletion, this creates an unstable near-equilibrium state (still having *z*-reflection symmetry) which releases in the course of time its energy into dipole oscillations between orbitals 1 and 2 (and between orbitals 6 and 7). Here, in the case of depletion by an XUV pulse, the system remains in a somewhat more shaken stage. But the initial dipole remaining right after the pulse is still very small such that the instability develops in the same manner as with an instantaneous depletion.

In the present paper, we aim to concentrate on the systematics of the growth time, $\tau$, and growth rate $1/\tau$. The heavy black line in Fig. 2 illustrates how we determine it quantitatively. We take two maxima of the dipole vector magnitude near the beginning, $t_1$, and end, $t_2$, of the region of pure exponential growth. The growth time is the time span over which the signal amplitude increases by factor *e*, i.e.

14 $$\begin{aligned} \tau = \frac{\log _e|\textbf{d}(t_2)|-\log _e|\textbf{d}(t_1)|}{t_2-t_1} \; \end{aligned}$$

equivalently, the gradient of the amplitude on the log scale. The depletion sampling time $t_s$ varies between separate calculations, and is chosen to be within the flat region of the $\overline{P}_1(t)$ curve, soon after the pulse has ended but before any further ionization occurs that is driven by the instability. Of course, the linear increase is never perfect due to unavoidable fluctuations of the signal (see time around 10 fs in Fig. 2). The growth time thus carries some uncertainty. We try to keep it small by taking a long interval (approximately 10 fs) in a region where the increase is most linear.

In the top panel, (a), the total post-pulse ionization decreases in the average as the laser frequency increases. This is the expected trend since the effective field strength decreases with increasing frequency for fixed intensity, according to the Keldysh formula^43^. Somewhat unexpected is the region of increasing ionization around $\omega _\textrm{pulse} =$ 52 eV. This coincides with a pronounced post-pulse population inversion $\Delta _{12}(t_s)$ (red line) which peaks at about $\omega _\textrm{pulse} =$ 55 eV. As argued in^21^, this is probably driven by a continuum resonance which preferably couples to orbitals 1 and 6. The growth rate $1/\tau$ (blue line) follows the trend of the population inversion which is plausible since the dipole instability is driven by the inversion.

### Dependence on pulse frequency

**Fig. 3:**
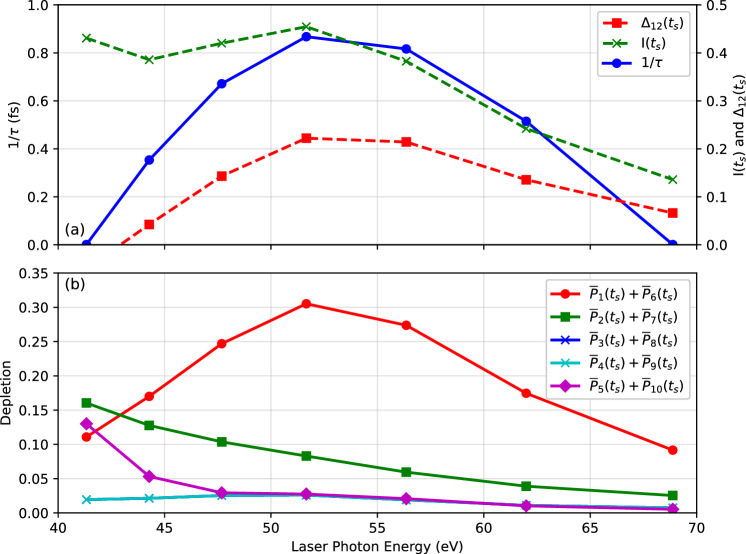
In upper panel (**a**), growth rate $1/\tau$ (blue solid line with circles), total ionization $I(t_s)$ (green dashes with crosses), and population inversion $\Delta _{12}(t_s)$ (red dashes with squares) as function of laser frequency after irradiation with laser pulses having a duration of $T_\textrm{pulse} =$ 1 fs and a peak intensity $I_\textrm{pulse}=$ $1\times 10^{15} \text{ W/cm}^{2}$. Both the laser polarization direction and the molecular axis are aligned along the *z*-axis. In lower panel (**b**), sum of Kohn-Sham orbital depletions $\overline{P}_n(t_s)$ for each pair of spin-degenerate orbitals. Calculations were performed using TM pseudopotentials in EDAMAME. $\Delta _{12}(t_s)$, $I(t_s)$ and $\overline{P}_j(t_s), j = 1,\dots , N_e$ are evaluated at appropriate depletion sampling time $t_s$ specific to each calculation, as described in Fig. 2.

Figure 3 displays the key observables as a function of the laser frequency after irradiation with laser pulses having a duration $T_\textrm{pulse} =$ 1 fs and a peak intensity $I_\textrm{pulse}=$ $1\times 10^{15} \text{ W/cm}^{2}$.

In Fig. 3b, we see the post-pulse depletions of each pair of spin-degenerate orbitals. We can see that orbitals 1 and 6 experience the strongest depletion at a photon energy around $\omega _\textrm{pulse} =$ 52 eV, in line with total ionization. The depletions of orbitals 2 and 7 decrease with increasing photon energy and become lower than the depletions of orbitals 1 and 6 at around $\omega _\textrm{pulse} =$ 43 eV. This indicates that the population inversion disappears below this value of photon energy, coinciding with the disappearance of the instability. At the higher end of the photon energy scale, population inversion is present, but the level of total ionization appears too low to drive the instability. Depletions of the other orbitals remain low enough to have no significant effect, over the range of photon energies considered.

Results are presented for the three different pseudopotentials (G, sG and TM) in order to study the effect of changing these. The total ionization $I(t_s)$ increases monotonically with intensity. In fact, it increases linearly as it should for one-photon processes. The population inversion $\Delta _{12}(t_2)$ follows the same trend as ionization, however, with a slight degrading for high intensities. For both these observables, the results obtained by the different pseudopotentials differ. In particular, the sG pseudopotential has generally much lower yields than the other two, even though the ionization potentials are comparable for all. The difference most probably comes from different high-momentum patterns of the pseudopotential. Fortunately, the differences are only quantitative while the overall trends remain similar.

### Dependence on pulse intensity

**Fig. 4:**
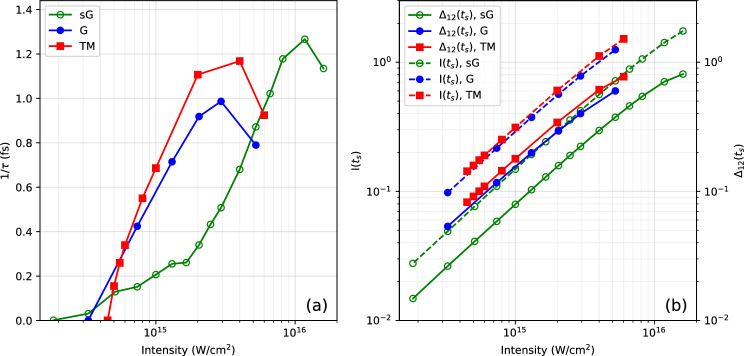
Growth rate $1/\tau$ (left panel) and total ionization $I(t_s)$ (right panel, full lines) as well as population inversion $\Delta _{12}(t_s)$ (right panel, dashed lines) as functions of laser intensity for a 1 fs pulse with frequency $\omega _\textrm{pulse}=59.04$ eV. Both the laser polarization direction and the molecular axis are aligned along the *z*-axis. Results are shown for the three pseudopotentials under consideration as indicated.

Now we pick a fixed laser frequency $\omega _\textrm{pulse}=59.04$ eV near the inversion maximum and study the response to varying the laser intensity. Figure 4 presents in the left panel the growth rate $1/\tau$, and in the right panel the population inversion $\Delta _{12}(t_s)$ and the total ionization $I(t_s)$ for a 1 fs pulse.

**Fig. 5:**
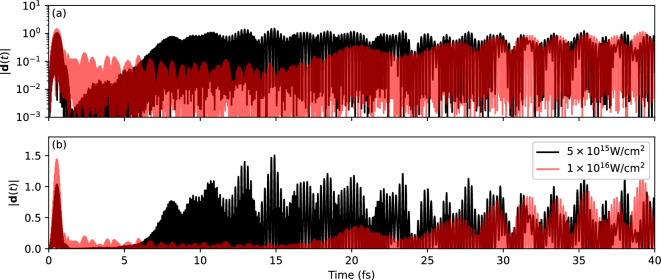
Time evolution of the dipole signal following an XUV pulse with frequency $\omega _\textrm{pulse}=59.04$ eV and pulse length of $T_\textrm{pulse} =$ 1 fs for two different intensities. Both the laser polarization direction and the molecular axis are aligned along the *z*-axis. Results were generated using the TM pseudopotentials in EDAMAME. The lower intensity $I_\textrm{pulse}=$ $5\times 10^{15} \text{ W/cm}^{2}$ corresponds to a case where the growth rate $1/\tau$ was decreasing but was still non-zero (see Fig. 3), while the higher intensity $I_\textrm{pulse}=$ $1\times 10^{16} \text{ W/cm}^{2}$ corresponds to a case where the growth rate could not be determined. Panel (**a**) shows the signal on a logarithmic scale and (**b**) in a linear scale.

The growth rates $1/\tau$ presented in Fig. 4 initially increase with laser intensity. These growth rates appear to peak at a value of around 1 fs$^{-1}$, with the intensity at which this occurs dependent on the pseudopotential used. The growth rates then decrease before the instability disappears altogether (at the point where all three curves terminate). This occurs in spite of the population inversion increasing over the whole intensity range. To understand what is happening in more detail, Fig. 5 presents the time evolution of the dipole signal $\left| \mathbf {d(t)}\right|$ calculated at two different laser intensities, one before (black lines) and one after (red lines) the instability disappears, according to Fig. 4. The lower intensity case shows the typical pattern of the dipole instability, namely an increase in the dipole signal that occurs after the laser pulse has ended. This signal eventually saturates at a non-zero value, as can be seen clearly on a logarithmic scale (upper panel). The higher intensity case differs already in the early phase in that the dipole signal does not decrease dramatically as the laser pulse ramps off. The stronger excitation has therefore stirred up the system much more, which is plausible. Surprisingly, the stronger initial dipole oscillations do not trigger an immediate further increase of the dipole signal. With a considerable delay, the dipole signal comes back up again. But it reaches at most the amplitude of the less highly excited case. All that indicates that a larger part of the initial excitation energy goes to other channels. The large residual dipole signal in the case of the stronger XUV excitation makes it very hard to track energy balance in detail.

Fortunately, we could also reproduce the disappearance of the dipole instability for the much cleaner excitation process of cutting an instantaneous hole from orbital 1. In the regime where the dipole instability disappears, we find instead a quadrupole instability from oscillations between orbitals 1 and 5 (both having positive *z*-parity). The large quadrupole oscillations, once established, pull, in turn, the dipole signal which then comes back with a large delay. That is what we also see in Fig. 5 for an XUV excitation. The detailed study of an instantaneous hole excitation is still ongoing. It goes beyond the scope of this paper and will be published later.

In previous explorations over a wide range of systems and conditions, we have encountered cases where the dipole instability does not show up at all, cases where it does not disappear once it was there, and here now a case of appearance and disappearance. After all, we see that the dipole instability results from a subtle interplay between system configuration, pulse profile, and, at a quantitative level, the pseudopotential parameters. Further investigations are required to develop simple rules for estimating when we can expect a well developed instability.

### Spectral properties of the dipole signal

**Fig. 6:**
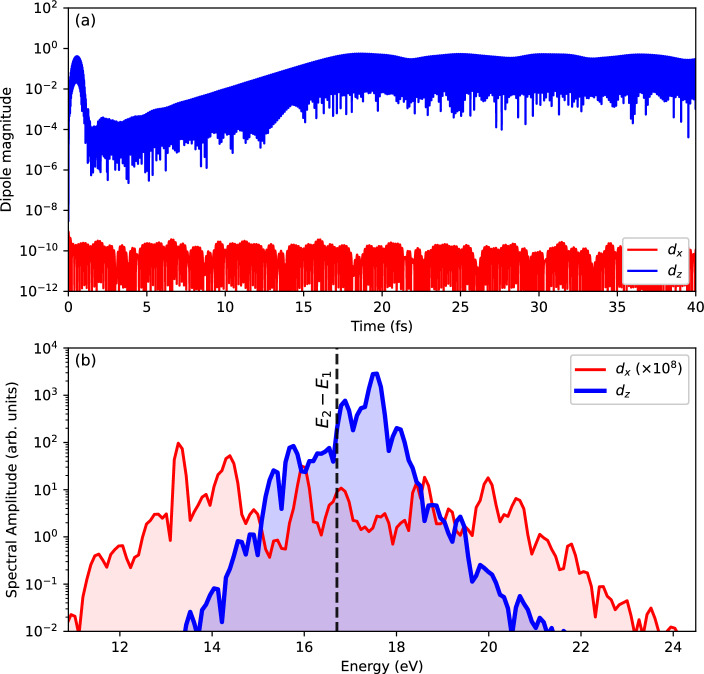
Dipole response of N$_2$ to a linearly polarized laser pulse of the duration $T_\textrm{pulse}= 1$ fs. The molecular axis is aligned along the *z*-axis. The laser pulse, linearly polarized and also aligned along the *z*-axis, and has a frequency $\omega _\textrm{XUV}=59.04$ eV and intensity $I_\textrm{pulse}=$ $8\times 10^{14} \text{ W/cm}^{2}$. The calculations were carried using TM pseudopotentials in EDAMAME. (**a**) Dipole signals (in a logarithmic scale) perpendicular to ($d_x$) and parallel to ($d_z$) the molecular axis in the time domain. We note that the other perpendicular component ($d_y$) responds in a similar manner to $d_x$. (**b**) Fourier transform of these dipole signals to the frequency domain. The Fourier transforms are windowed over the time window $t=0-48.4$ fs. The transform in the perpendicular direction has been scaled by a factor of $10^{8}$ to allow for an easier visual comparison. The vertical dashed line corresponds to the difference in the single particle energy difference of orbitals 1 and 2.

Figure 6 analyses the spectral composition of the dipole signal in both directions, parallel (along the *z*-axis) and perpendicular (along the *x*-axis) to the molecular axis. In this case a linearly polarized laser pulse of the duration $T_\textrm{pulse}= 1$ fs was considered, having a frequency $\omega _\textrm{XUV}=59.04$ eV and intensity $I_\textrm{pulse}=$ $8\times 10^{14} \text{ W/cm}^{2}$. The calculations were carried using TM pseudopotentials in EDAMAME. Panel (a) shows the dipole signals in the time domain. In the parallel direction, we see the signature of the dipole instability. Practically no signal appears in the perpendicular direction in spite of the fact that the occupied states of N$_2$ also offer a pronounced dipole transition in that direction, namely between orbitals 1 and orbitals 3 and 4. The soaking power of the orbital 1 to orbitals 3 and 4 mode seems to be stronger. Dipole spectra in panel (b) thus show only weak noise for the perpendicular direction. The parallel direction has a distinguished peak near the orbital 1 to 2 transition energy. The slight energy shift is due to the residual Coulomb interaction.

**Fig. 7:**
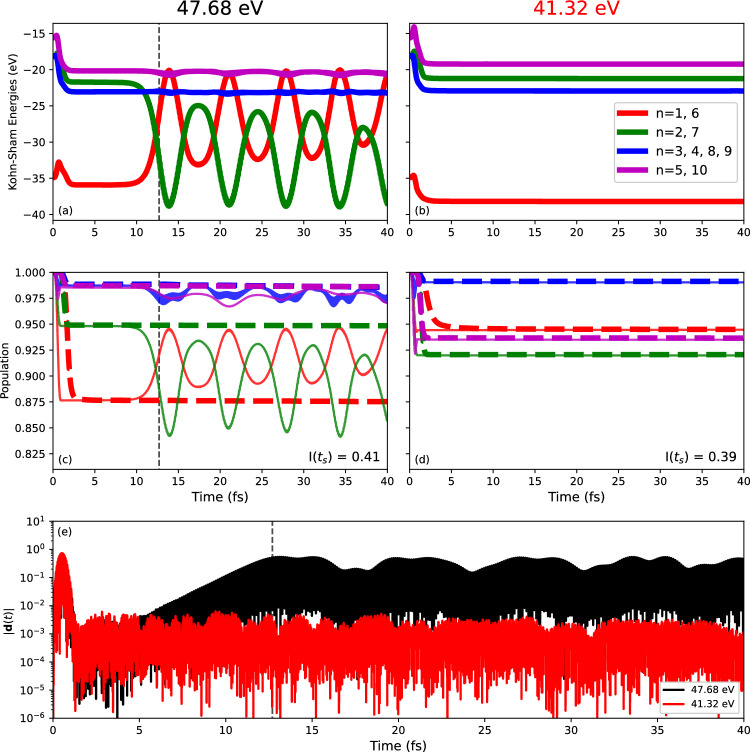
Time evolution of single particle properties of N$_2$ after XUV excitation by a laser pulse with duration $T_\textrm{pulse}= 1$ fs and intensity $I_\textrm{pulse}=$ $1\times 10^{15} \text{ W/cm}^{2}$. Two laser frequencies were considered: $\omega _\textrm{pulse}=47.68$ eV and $\omega _\textrm{pulse}=41.32$ eV. Both the laser polarization direction and the molecular axis are aligned along the *z*-axis. (**a**,**b**) Show the time-dependent Kohn-Sham energies for each occupied Kohn-Sham orbital. The full lines in (**c**,**d**) show the initial orbital overlaps $S_n(t), n = 1, \dots ,N_e$ while the dashed lines show the populations ${P}_j(t), j = 1, \dots ,N_e$. The left panels, (**a**,**c**), present results for the higher frequency, while the right panels, (**b**,**d**), present results for the lower frequency. (**e**) Shows the time-dependent dipole signals for both frequencies. The calculations were performed using TM pseudopotentials in EDAMAME. The dashed black lines in (**a**,**c**,**e**) at $t=13$ fs denote the point at which the dipole signal saturates for the higher frequency pulse.

**Fig. 8:**
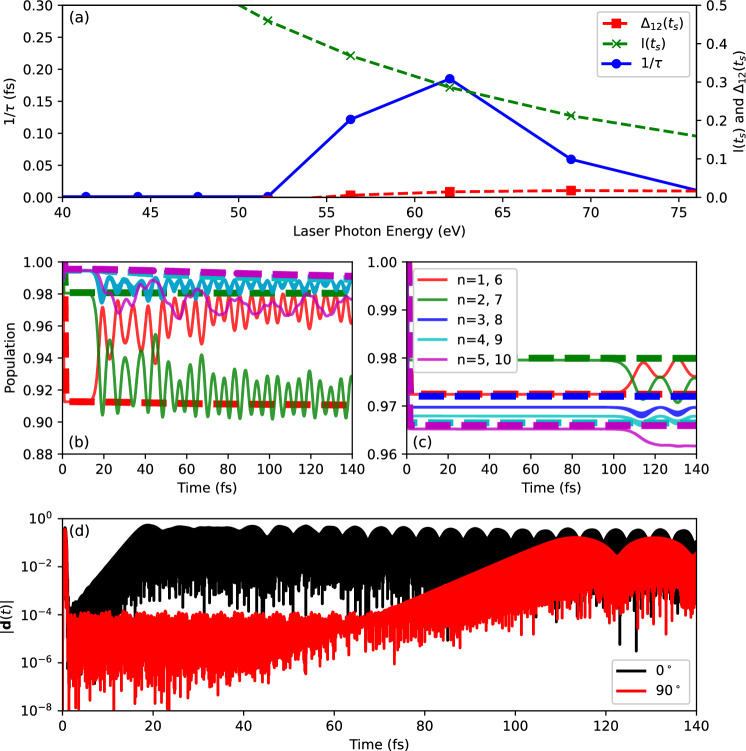
In upper panel (**a**), growth rate $1/\tau$ (blue solid line with circles), total ionization $I(t_s)$ (green dashes with crosses), and population inversion $\Delta _{12}(t_s)$ (red dashes with squares) as function of laser frequency after irradiation with laser pulses having a duration of $T_\textrm{pulse} =$ 1 fs and a peak intensity $I_\textrm{pulse}=$ $1\times 10^{15} \text{ W/cm}^{2}$. The laser polarization direction is along the *z*-axis while the molecular axis are aligned along the *y*-axis. The full lines in panels (**b**,**c**) show the initial orbital overlaps $S_n(t), n = 1, \dots ,N_e$ while the dashed lines show the populations ${P}_j(t), j = 1, \dots ,N_e$ when considering one particular laser frequency: $\omega _\textrm{pulse}=61.99$ eV. For (**b**) both the laser polarization direction and the molecular axis are aligned along the *z*-axis (0° orientation). For panel (c) the laser polarization direction is along the *z*-axis while the molecular axis are aligned along the *y*-axis (90° orientation). (**e**) Shows the time-dependent dipole signals for both orientations considered in (**b**,**c**). The calculations were performed using TM pseudopotentials in EDAMAME.

### Details of the interplay between orbitals 1 and 2

For a more detailed overview of the single particle dynamics, we present in Fig. 7 the time evolution of dipoles, single particle energies, populations $P_j(t), j = 1, \dots ,N_e$ and initial orbital overlaps $S_n(t), n = 1, \dots ,N_e$ for two laser frequencies: $\omega _\textrm{pulse}=47.68$ eV and $\omega _\textrm{pulse}=41.32$ eV. In both cases the laser pulse has duration $T_\textrm{pulse}= 1$ fs and intensity $I_\textrm{pulse}=$ $1\times 10^{15} \text{ W/cm}^{2}$. The lower frequency does not trigger a dipole instability while the higher frequency does, as seen from the dipole signal in panel (e). In the higher frequency case, we see that the dipole signal saturates at around 13 fs. The single particle energies, panels (a) and (b), decrease after the passage of the laser pulse due to charging of the system by ionization. They remain approximately constant afterwards. However, in the higher frequency case in panel (a), the single particle energies for orbitals 1 and 2 begin oscillating at around 13 fs indicating that the instability is related to mixing of these two orbitals. This interpretation is corroborated by the initial orbital overlaps in panels (c) and (d). In the case where the dipole instability occurs, panel (c), similar oscillations in the initial orbital overlaps occur at the same point as the oscillations in the single particle energies. While these oscillations in the single particle energies and initial orbital overlaps occur, the orbital populations (dashed lines) show no such oscillations.

In addition, Fig. 7c, we see that the initial orbital overlaps of orbitals 1 and 2 begin to deviate from their respective populations during the exponential increase in the dipole signal. These two overlaps then oscillate in anti-phase with one another following the saturation of the dipole signal at around 13 fs. This behaviour, i.e. the deviation and oscillation, can be understood as a change in character of the Kohn-Sham orbitals as we propagate in time, i.e. the time-evolving orbitals 1 and 2 grow into a coherent superposition of both. In general a superposition of two states that have a non-zero transition dipole and a non-zero energy difference will lead to an interference oscillation that grows in amplitude as the state mixing increases^44^. This oscillation would be expected to occur at close to the single particle energy difference of the two states, and with a polarization in the direction of their transition dipole. This turns out to be consistent with the result shown in Fig. 6b. There is a small side-effect, though, to the extent that orbitals $n=3$, 4 and 5 are also catching some small oscillations due to the oscillations of the mean field.

### Orientation effects

In realistic experiments, a large sample of molecules having different orientations to the laser polarization direction will be present (depending on the amount of alignment), with each molecule experiencing different laser intensities in the focal volume. The interaction of all these dipoles as they propagate through the medium can obviously have a large influence on the radiation detected experimentally ^45,46^. While studying these macroscopic propagation effects is beyond the realm of this paper, we can say more about how the instability changes as we vary the intensity and orientation. Indeed, Fig. 4 already shows how the instability changes as the laser intensity is varied.

So far we have only considered linearly polarized laser pulses aligned parallel to the molecular axis. However, it is well-known that when considering High-Harmonic Generation (HHG) in linear molecules like N_2_, orientation effects can have a huge influence on the measured harmonic spectra. For example, it was shown ^47^ that the cut-off harmonics were enhanced in the perpendicular alignment compared to the parallel case. In that case the HOMO does not respond predominantly to the field in the perpendicular orientation compared to the more deeply bound HOMO$-1$. This disputed behavior  ^48^ turns out to be robust against macroscopic propagation effects ^46^.

It is thus interesting to explore how the instability behaves as we vary the angle between the laser polarization direction and the molecular axis in this work. Keeping the laser polarization direction along the *z*-axis, we now consider the perpendicular orientation case where the molecule is aligned along the *y*-axis. Using the same laser parameters considered in Fig. 3, Fig. 8a presents the growth rate $1/\tau$ (blue solid line with circles), total ionization $I(t_s)$ (green dashes with crosses), and population inversion $\Delta _{12}(t_s)$ (red dashes with squares) as function of laser frequency. For space reasons, we will not present the sum of Kohn-Sham orbital depletions $\overline{P}_n(t_s)$ for each pair of spin-degenerate orbitals.

Looking at the growth rate, we see that the instability does appear. However, the frequency range over which the instability appears is different for the parallel alignment as shown in Fig. 3a. Not only is the frequency window for the instability narrower, it is at higher photon energies. In addition, the growth rates are smaller than in the parallel alignment. In our previous study of acetylene ^22^, we considered one particular laser pulse and showed that while the instability was present for the parallel alignment, it was not present in the perpendicular alignment. Our results here show that there may be instances where the instability is present in acetylene in the perpendicular alignment.

Let us now consider a case where the instability should be present in the perpendicular alignment, namely for a laser pulse having a frequency $\omega _\textrm{XUV}=59.04$ eV and an intensity $I_\textrm{pulse}=$ $1\times 10^{15} \text{ W/cm}^{2}$. Figure 8b,c present the initial orbital overlaps and populations for the parallel and perpendicular alignments respectively, while (d) shows the time-dependent dipole signals. We see that the instability is present in both cases but that the growth rate is lower in the perpendicular orientation. We see that the initial orbital overlaps of orbitals 1 and 2 in panels of Fig. 8 show a similar behaviour to what was observed in Fig. 7c.

In both these alignments, we are dealing with an effect involving a transition between the same two states. Hence, we would expect that if macroscopic propagation effects were included, the dipole instability should still manifest itself. But the amplitude of the signal, at a given intensity, may be reduced. In addition, it may as well be difficult to distinguish particular features in the spectrum of light (Fig. 6b, for example).

## Conclusion

In this paper, we have continued our investigations of a dipole instability mechanism observed in small molecules and clusters under particular irradiation conditions. These conditions especially cover ultrafast scenarios for which a description in terms of instantaneous depletion of deeply bound single particle levels is frequently used. In earlier publications^21,22^ , we found that one can drive XUV laser pulses in a way which also generates such a depletion. We continued on that line and presented here the test case of N$_2$ explicitly irradiated by ultrafast laser pulses (1 fs duration). We mention in passing that we also looked at longer pulses as well as at other systems and found similar effects. The aim here is to explore timescales and the onset of instability when varying laser intensity, frequency and orientation.

One focus of the paper was to examine the impact of the physical description of the system, especially in terms of the treatment of electron-ion interactions. Our former papers on the topic^20–22^ have already addressed pure electronic aspects both at the formal and practical levels. The dipole instability is an exponential decay of the excitation from occupation inversion accompanied by exponential increase of the dipole signal. A possible competitor is the decay of the excitation by dissipation. Estimating dissipation by a relaxation-time approximation^30^, we have shown earlier that, in the considered cases, dissipation does not overrule the observed instability^21,22^. From a practical viewpoint, extensive numerical checks and use of two totally independent real-time TDDFT packages, namely QDD^21^ and EDAMAME^22^, have demonstrated the robustness of the instability behavior. The question of whether the effect is a consequence of a TDDFT treatment nevertheless remains open, as well as its possible experimental identification via time-resolved Photo-Electron Spectra^21^.

Our former investigations have shown that the appearance of the instability is correlated to a population inversion, which nevertheless, at least in the explored cases, could not be traced back to an Auger effect for energetic reasons. Another focus of the present paper was thus to refine the analysis of this population inversion. We have identified a clear oscillation of population between two single particle energy levels coupled by a sizable dipole transition. This reinforces the role of the population inversion already identified earlier and gives it a more quantitative status by identification of well defined transitions between single particle levels.

A key quantity characterizing the instability is the rate of growth thereof. We have thus extensively studied its behavior as a function of laser parameters. It is also extremely telling to correlate these trends with the total ionization in terms of the population inversion reflecting the transition between two specific single particle levels. Qualitatively, and to some extent quantitatively, the growth rate increases with excitation, i.e. occupation inversion, and is independent of the pseudopotential used for describing electron-ion interactions. The same holds true for the total ionization and population inversion. The present study revealed a new aspect which we had not seen before. There are cases where the growth rate of the dipole instability turns to a decrease with increasing pulse intensity. The reason for that could be related to a competition with a quadrupole instability which takes over for high excitation energies.

The occupation inversion has been further investigated in some detail. The spectral analysis of the dipole, in particular, very clearly exhibits a peak corresponding to the involved single particle level transition. The population inversion thus seems rather well established in that case.

The microscopic mechanisms beyond the instability remain to be further investigated. In particular, a quantitative estimate of the conditions for the instability and the dipole/quadrupole competition has yet to be developed. The present paper has brought several interesting results, confirming the existence and the robustness of this instability and the associated mechanism. This provides a clue to search for similar situations (population inversion) in the other cases we have investigated and to work out a generic scenario explaining the instability, by considering simplified model systems.

It has also been observed that similar instabilities occur when considering instantaneous excitations of 1D atomic wires described using self-consistent tight-binding (SCTB) models of electronic structure ^49^. Other polarizable SCTB descriptions of instantaneous excitations in molecules such as azulene have also reported ring currents that appear after several hundred femtoseconds ^50^. We are currently investigating whether these observations are related to the dipole instabilities.

## Data Availability

The datasets generated during the current study are available. 10.17034/8196d135-92b9-4ef9-bab8-49c90640a2b8.
